# Medical Education in the United States

**Published:** 1911-01-07

**Authors:** 


### The Hospital

A Journal of

The Medical Sciences and Hospital Administration.

New Series. No. 205, Vol. VIII. [No. 1277, Vol. XLIX.] SATURDAY, JANUARY 7, 1911.

### MEDICAL EDUCATION IN THE UNITED STATES.

In the United Kingdom we are familiar with many intricate problems concerning medical education, medical degrees, medical schools, and cognate matters; and widely diverse opinions are held as to the best methods of solving admitted defects in the present systems, and even as to whether some characteristics are defects at all. In the United States there are problems far graver and more imperative than any which beset us, if a correct judgment can be formed from the Report to the Carnegie Foundation which has been presented by Dr. Abraham Flexner. The bogus "colleges" and "universities" which once trafficked freely in medical degrees, and would confer the status of M.D. upon anyone prepared to pay five or ten pounds for that distinction, are by this time mostly suppressed; though even now there must be many curiously conducted concerns such as that "homœopathic hospital" which conferred its M.D. upon Hawley Crippen without requiring of him any training in practical surgery. But if that disgraceful page of American medical history has been turned down, it is yet true that the laxity in regulating those slightly more pretentious and slightly more real medical schools which still flourish is such as to call forth the severest strictures from the Carnegie Commissioner. The attitude of the Foundation is that all colleges and universities, whether supported by taxation or by private endowment, are in truth public service corporations; and that the public is entitled to know the facts concerning their administration and development, both on the financial and educational sides. It believes, therefore, that in seeking to present a fair and accurate statement of the work and the facilities of the medical schools of the United States, it is serving the best possible purpose which such an agency can serve; and that only by such publicity can the true interests of education and of the universities be furthered. In such a reasonable publicity lies the hope of progress in medical education.* The Flexner report has excited enormous interest in the United States; every medical and every other lay journal there devotes much space to its discussion.

For twenty-five years past, according to the Report, there has been in America an enormous over-production of untrained and ill-trained medical practitioners. This has been in absolute disregard of the public welfare, and without any serious thought of the interests of the public. In the States, as a whole, physicians are four or five times as numerous in proportion to population as in Germany. The over-production of ill-trained men is due in the main to a very large number of commercial schools, sustained in many cases by advertising methods whereby a mass of unprepared youth is drawn out of industrial occupations into the study of medicine. Until recently the conduct of a medical school, often entirely devoid of facilities for clinical teaching such as are afforded by a hospital, has been a profitable business, for the methods of instruction were mainly didactic. But the need for expensive laboratories and the other elaborate environment of the modern medical student has resulted in a deterioration in the standard of instruction which the small schools can offer. Nearly half the medical schools in America, it seems, have incomes of less than £2,000 a year.

Very variable and almost nebulous are the relations between colleges and universities, and between either of them and the hospitals. In some cases the connection is little more than a licence from a university by which a proprietary medical school is able to assume its name. In other cases the medical school is incorporated into the college or university, but remains a distinct entity, the college assuming no responsibility for its standards or its support; and sometimes there is a financial obligation without any corresponding control over the teaching or general educational efficiency. But seldom is there to be found a medical school as an integral part of a university, receiving from it university standards and adequate maintenance. The existence of many of these unnecessary and inadequate medical schools has been defended on the ground that they are in the interests of the poor scholar; but the report makes it evident that this argument is insincere, and that the excuse which has been put forward in the name of the poor boy is in reality an argument on behalf of an inefficient school. Not in this way is sound medical education in Scotland placed within the reach of the indigent student, provided he be steady and hard working.

Progress for the future demands a reduction in the number of medical schools, with a levelling up in their equipment and their curriculum. Equally it is desirable that fewer physicians should become qualified each year, but that those who do qualify shall be better instructed. In future the college or university which accepts a medical school must make itself responsible for university standards in the medical school and for adequate support for medical education. So says the Carnegie Foundation Report; and in view of the serious state of affairs therein disclosed it is impossible not to agree. Furthermore, the fact that in Great Britain matters have never come to quite such a pass must be ascribed to the influence of that much-abused body, the General Medical Council. If such a body existed in the United States, properly fulfilling the duties of inspection at examinations such as the licensing corporations here have to submit to, it is inconceivable that medical education could have been thus commercialised and degraded. We in Britain have many beams to remove from our own professional vision; but even with those hindrances to correct judgment it does seem as if there is much to be thankful for which is not done better across the Atlantic. The suggestion may be hazarded that the success of post-graduate courses in America may be partly due to inadequate pre-graduate training.

* *American Journal of Obstetrics and Gynæcology*, October 1910.

